# Combination of Arsenic trioxide and Everolimus (Rad001) synergistically induces both autophagy and apoptosis in prostate cancer cells

**DOI:** 10.18632/oncotarget.14493

**Published:** 2017-01-04

**Authors:** Sheng Tai, Lingfan Xu, Ming Xu, Ligang Zhang, Yangyang Zhang, Kaipin Zhang, Li Zhang, Chaozhao Liang

**Affiliations:** ^1^ The Department of Urology, The First Affiliated Hospital of Anhui Medical University, China; ^2^ The Department of Urology, The Urological Institute of Anhui Medical University, China

**Keywords:** Rad001, ATO, synergism, autophagy, prostate cancer

## Abstract

The inhibitor of PI3K-AKT-mTOR pathway, such as Rad001, has not shown therapeutic efficacy as a single agent in prostate cancer. Arsenic trioxide induces the autophagic pathway in prostate cancer cells. We identified Arsenic trioxide can synergize with Rad001 to induce cytotoxicity of prostate cancer cells. Moreover, we identified synergistic induction of autophagy and apoptosis as the underlying mechanism. This enhanced autophagic cell death is accompanied by increased Beclin1 mRNA stability as well as upregulation of ATG5-ATG12 conjugate, *Beclin1*, and LC3-2. Rad001 and ATO also can synergistically inhibit tumors in prostate cancer xenograft animal model. These results identify and validate a novel mechanism to enhance and expand the existing targeted therapeutic agent to treat prostate cancer.

## INTRODUCTION

Prostate cancer (PCa) is the most common cause of cancer-related death in the United States [1–3]. The early stages PCa may be cured by certain therapeutic strategies such as radical surgery or radiation, but the therapeutic effect for advanced and metastatic PCa is poor [4, 5]. It has been reported that most PCa may evolve into the castration resistant prostate cancer (CRPC) if the patients with PCa underwent the castration [6, 7], whose mechanisms may be attributable to the resistance to Androgen Receptor (AR), increased expression of AR, AR mutations/phosphorylation/splice variants [6]. Therefore, it is necessary to develop and explore the effective and novel therapies. It has been reported that the PI3K-AKT-mTOR pathway is vital of importance for the PCa progression [8, 9].

**Figure 1:**
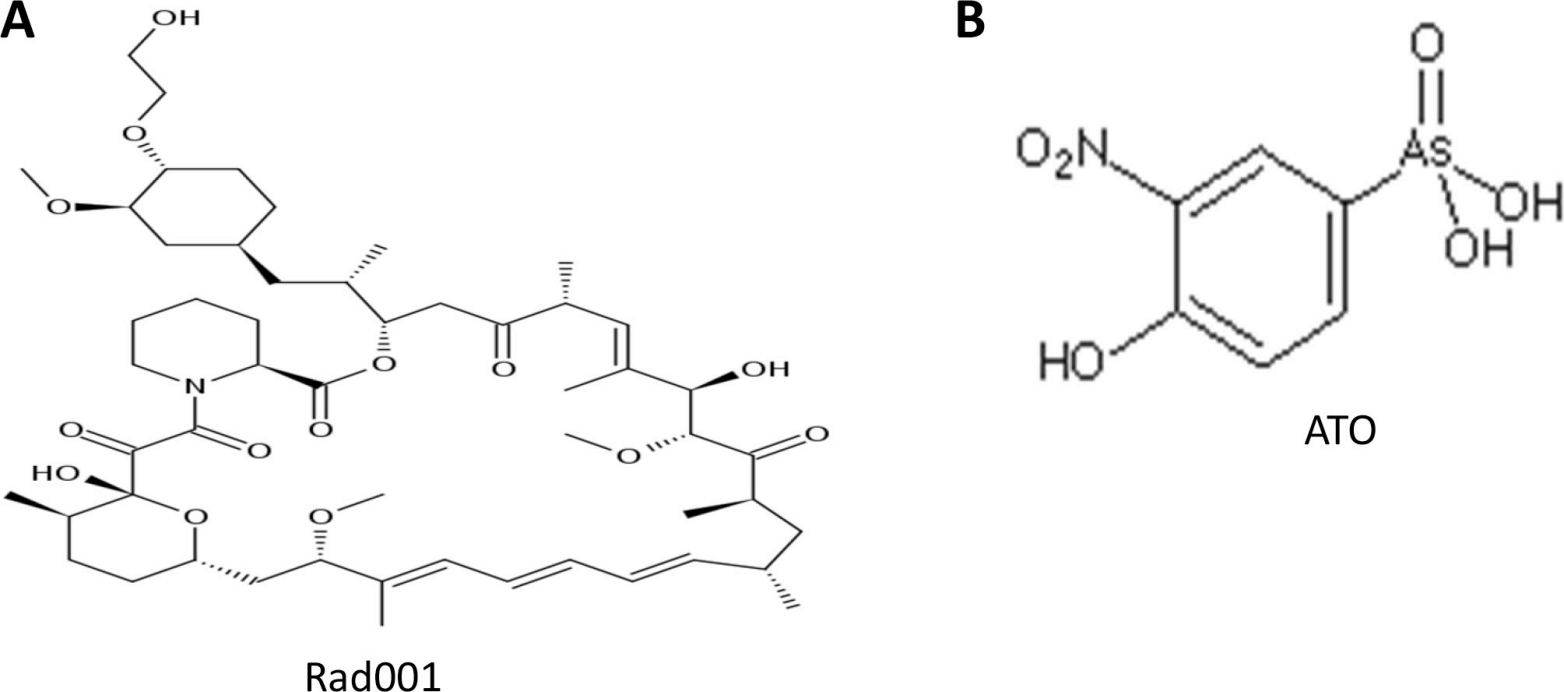
The chemical structures of Rad001 and ATO The structure of Rad001 was presented in the Figure 1 (**A**) Figure 1 (**B**) showed the chemical structure of ATO.

The Rad001 (Figure 1A) is one of the mTOR, the PI3K-AKT-mTOR pathway, specific inhibitors, which may exert a negative effect on the PCa progression. However, its therapeutic effect in the PCa is poor, which may be attributable to activation of other bypass signaling pathways [10]. The Arsenic trioxide (ATO, Figure 1B) has been administered by the FDA to treat the acute promyelocytic leukemia (APL) [11, 12]. Certain studies showed a lot of solid tumors proliferation may be suppressed with ATO treatment [13, 14]. However, its severe side effects, hepatic toxicity, cardiac toxicity, granulocytopenia, etc, may confine it therapeutic effect in clinical practice.

In order to probe into the molecular mechanisms of cell death, more and more studies have focused on the autophagy and apoptosis. The autophagy is characteristic of abundant autphagic vacuoles in the cytoplasm [15, 16]. The inhibition of PI3K-AKT-mTOR pathway may induce the autophagic cell death [16]. In the present study, we probed into how the combination of Rad001 and ATO synergistically induced the prostate cancer cells death and inhibit the prostate cancer xenograft tumor proliferation. We found that the combination treatment may induce both autophagy and apoptosis in prostatic LNCaP and PC3 cell lines. The LNCaP cells expressed the prostatic specific antigen (PSA) and androgen receptor (AR), with features characteristic of prostatic adenocarcinoma [17]. On the other hand, the PC3 cells are negative for AR and PSA but are positive for neuroendocrine markers, similar to human prostate small cell carcinoma, representative of the most late stage or aggressive prostate cancer [18]. The LNCaP and PC3 prostate cancer cell lines appear to have different sensitivities to the combinatorial treatment, which may have clinical implications in the future studies. More importantly, the combination of ATO and Rad001 can also synergistically suppress xenograft model tumors growth. Our studies suggest a seminal potential therapeutic strategy for advanced and recurrence PCa in the future.

## RESULTS

### Bright filed images of cells and matrigel colony formation assay

**Figure 2:**
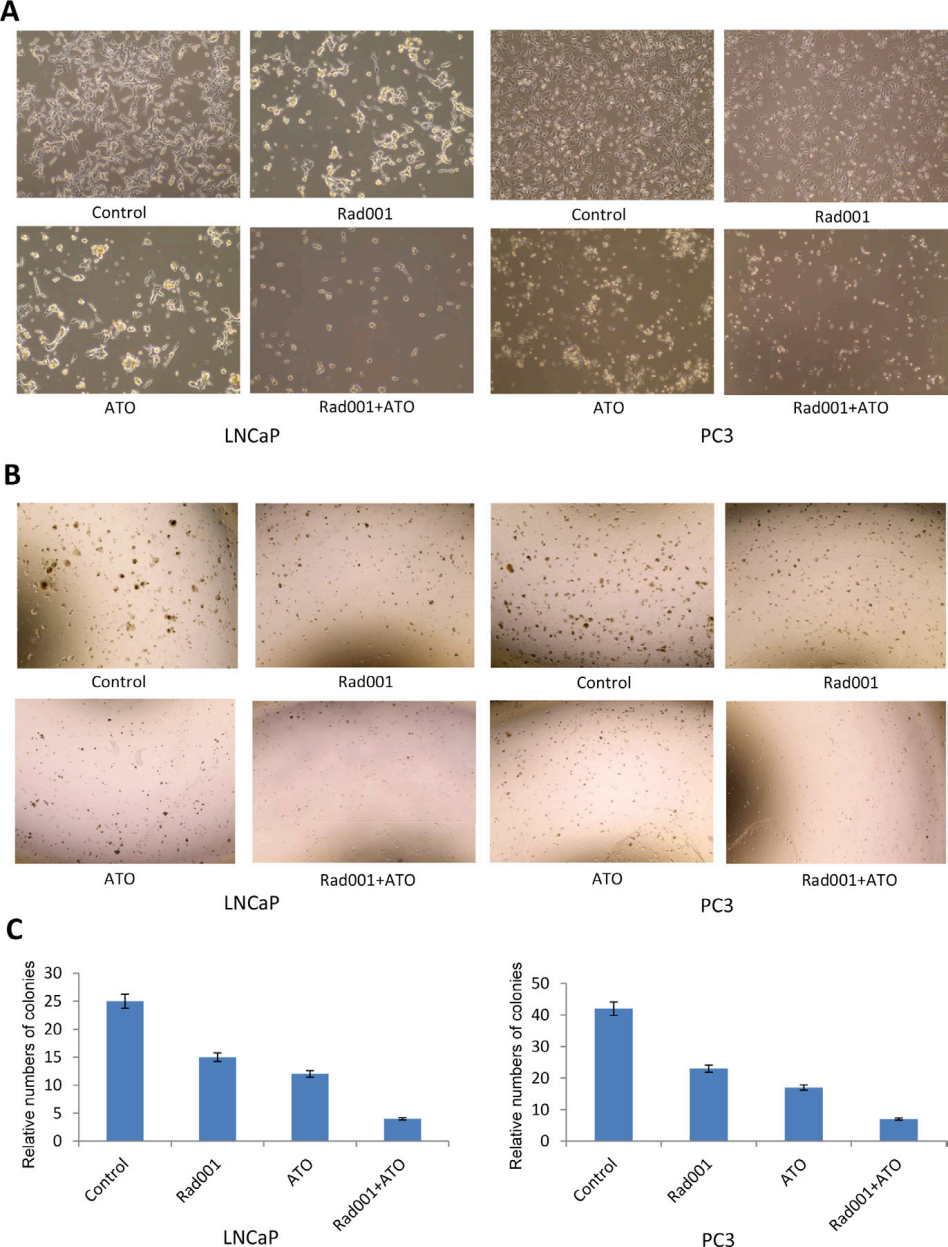
The combination of Rad001 and ATO can synergistically inhibit the colony formation in prostate cancer cells For LNCaP cells, they were treated DMSO (control), Rad001 (0.37 μM), ATO (11.19 μM), and their combination, for 24 hrs. For PC3 cells, they were treated DMSO (control), Rad001 (0.35 μM), ATO (12.00 μM), and their combination, for 24 hrs. The cell morphology was detected with the microscopy (**A**). After the prostate cancer cells treated DMSO (control), Rad001, ATO, and their combination for 14 days, the colony formation was detected (**B**), and the relative number of colony formation was quantified (**C**).

The combination of ATO and Rad001 can synergize prostate cancer LNCaP and PC3 cells morphologic alterations, compared with alone treatment (Figure 2A). The colony formation ability in prostate cancer LNCaP and PC3 cells treated with two compounds was significant inhibited at the 14^th^ day, compared with one compound treatment (Figure 2B), suggesting the prostate cancer cells tumorigenesis may be synergistically inhibited with the combination of ATO and Rad001 treatment. The quantification of colony formation is shown in Figure 2C.

### Combination of ATO and Rad001 synergistically inhibiting the LNCaP and PC3 prostate cancer cell lines

**Figure 3:**
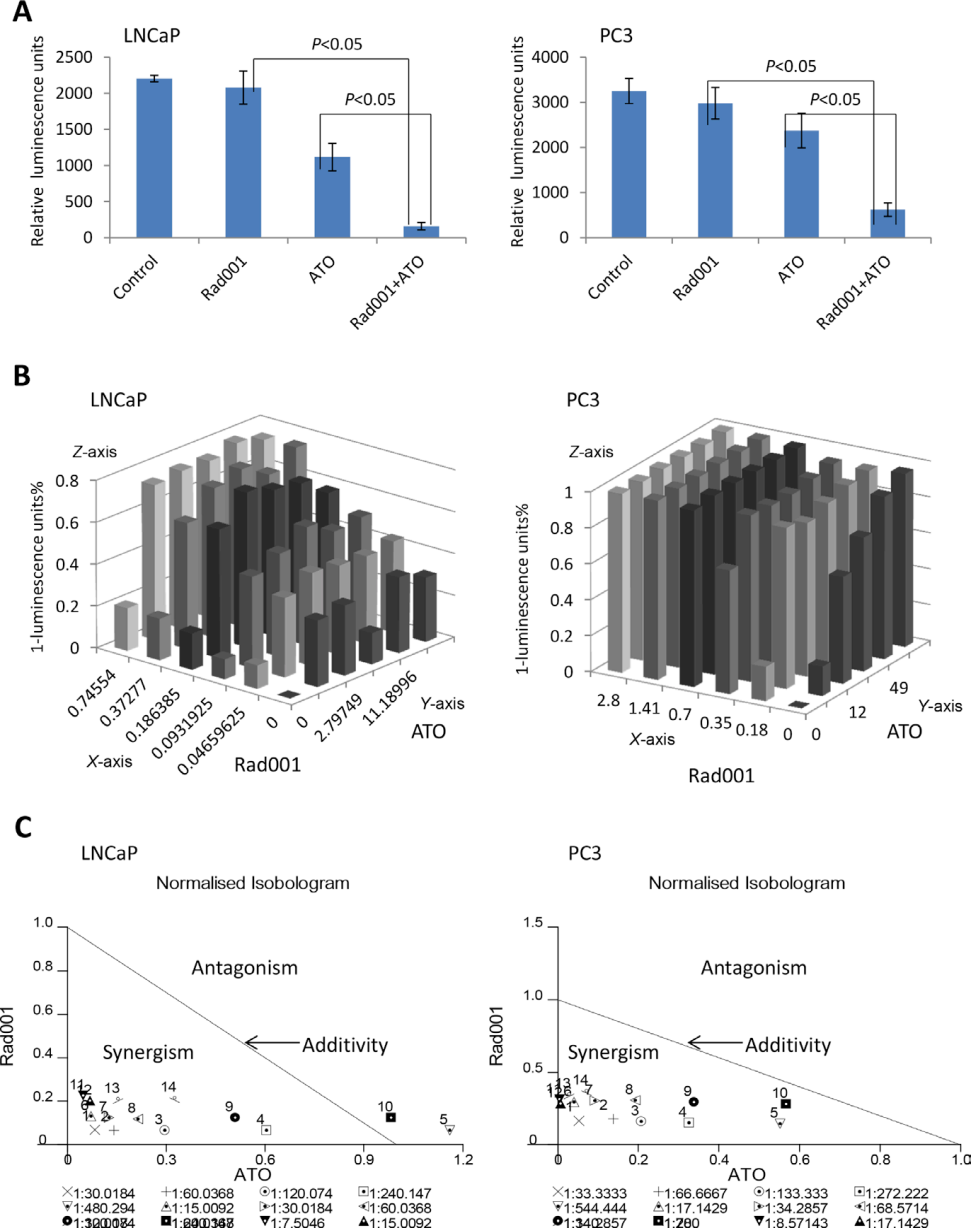
The combination of Rad001 and ATO induced a synergistic reduction of cell number in prostate cancer cells The ATO can synergize with Rad001 to inhibit cell luminescence units in prostate cancer LNCaP and PC3 cells. For LNCaP cells, they were treated DMSO (control), Rad001 (0.37 μM), ATO (11.19 μM), and their combination, for 24 hrs. For PC3 cells, they were treated DMSO (control), Rad001 (0.35 μM), ATO (12.00 μM), and their combination, for 24 hrs. The cell survival was detected with Celltiter Glo measurement (**A**). (**B**) A gradient dose of Rad001, ATO alone and the combination was used to detect cell survival as in Figure 3B (*X*-axis: Rad001 μM, *Y*-axis: ATO μM, *Z*-axis: 1-luminescence-units%). (**C**) Normalised isobologram analysis showed synergistic interactions in prostate cancer LNCaP and PC3 cell lines. Normalised isobologram assays were achieved with *CalcuSyn* software, which can perform the drug does-effect calculation with the median effect method described by Chou TC (34). A large number of combination groups were below the line, indicative of synergism, in the prostate cancer cells.

To confirm that Rad001 and ATO can work synergistically to reduce the cell luminescence units, we added each of the two compounds at either gradient concentrations, and detected their effects on the luminescence units of LNCaP and PC3 cells. The Figure 3B showed that the dose-dependent luminescence units of prostate LNCaP and PC3 cells were detected, when the cells were administered with ATO or Rad001 alone. The ATO ED_50_ of LNCaP cell lines was 22.38 μM, and the ED_50_ for Rad001 was 0.75 μM. For PC3 cells, the ED_50_ for ATO and Rad001 were 24.45 μM and 0.70 μM, respectively. However, for both PC3 and LNCaP prostate cancer cell lines, the combination of ATO and Rad001 treatment resulted in more reduction of cell luminescent units (*Z*-axis), compared with either ATO or Rad001 alone treatment (Figure 3B). There was the significant synergism of the combination of ATO and Rad001, according to the combination index (CI) determined with the *CalcuSyn* program (Figure 3C). When the LNCaP cells were treated with 0.37 μM Rad001, the cell luminescence units reduced to 13.62%; and 11.19 μM ATO treatment alone induced 32.23% reduction in cell luminescence units. However, the reduction of cell luminescence units increased to 68.29% (CI = 0.76) when treatment with two compounds. On the other hand, when the PC3 cells were treated with 0.18 μM Rad001, the cell luminescence units reduced to 15.67%; and 6 μM ATO treatment alone induced 18.67% reduction in cell luminescence units. However, the reduction of cell luminescence units increased to 78.12% (CI = 0.57) when treatment with two compounds.

According to Mixture–Algebraic analysis, the combinations of two compounds with the wide range of combinatorial concentrations have the synergism, suggesting a large number of combination groups had the synergistic reduction of cell luminescence units. According to results of *CalcuSyn* software, we chose different drug concentration for LNCaP and PC3 cell lines. The LNCaP cell lines were treated with DMSO, 0.37 μM Rad001, 11.19 μM ATO and combinatorial ATO and Rad001 for 24 hrs. The PC3 cell lines were treated with DMSO, 0.35 μM Rad001, 12.00 μM ATO and combinatorial ATO and Rad001 for 24 hrs. We performed luminescence units of the cells after the drug treatment, there was a significant decrease luminescence units in combinatorial group compared with alone compound group in both LNCaP and PC3 cell lines (Figure 3A), suggesting the combination of ATO and Rad001 treatment led to more reduction of luminescence units. Later trials are according to above the drug concentrations.

### Combination of ATO and Rad001 synergistically induced apoptosis cell death in prostate cancer LNCaP and PC3 cell lines

**Figure 4:**
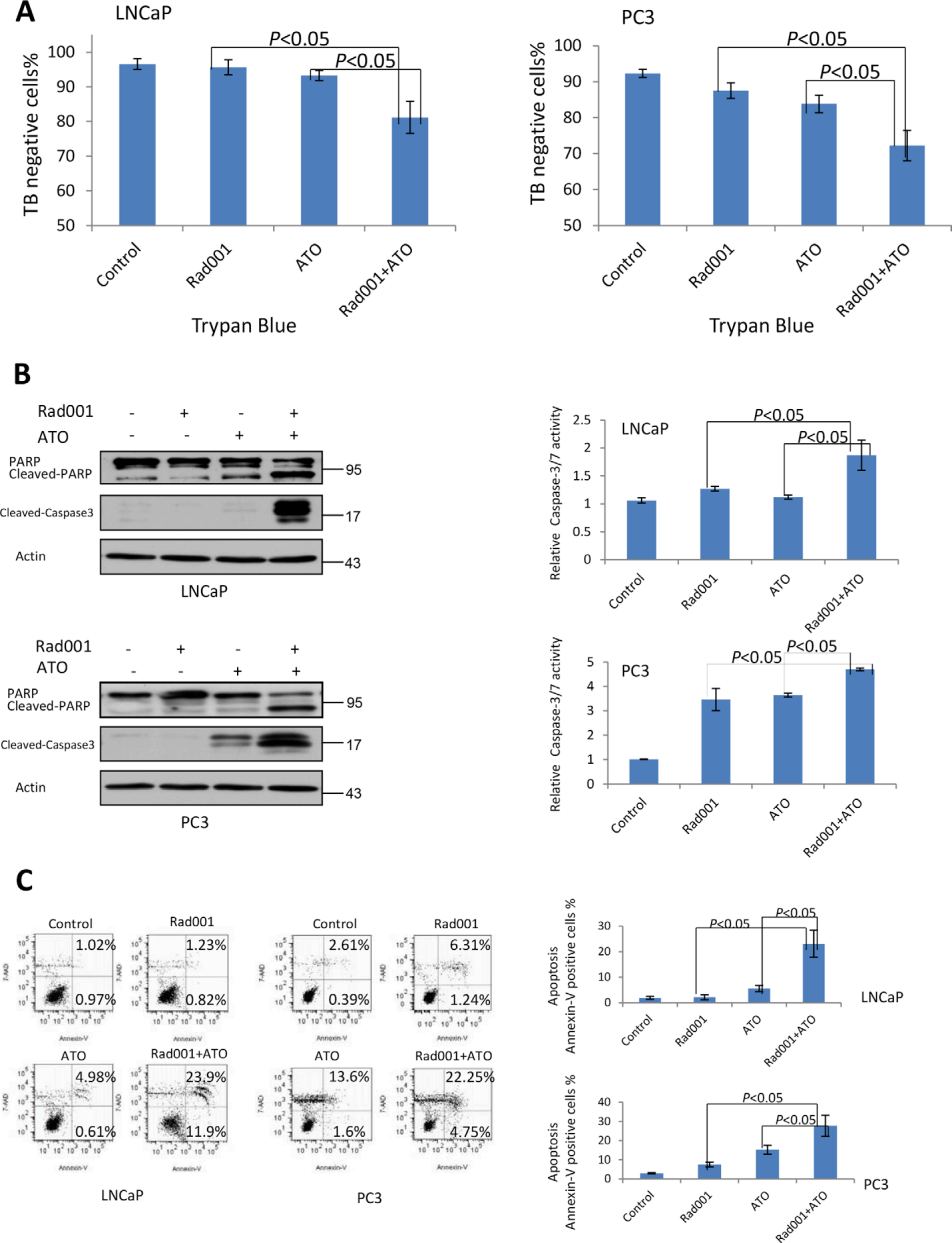
Rad001 and ATO combination synergistically induced cell death in prostate cancer cells For LNCaP cells, they were treated DMSO (control), Rad001 (0.37 μM), ATO (11.19 μM), and their combination, for 24 hrs. For PC3 cells, they were treated DMSO (control), Rad001 (0.35 μM), ATO (12.00 μM), and their combination, for 24 hrs. (**A**) Trypan blue (TB) analysis of the LNCaP and PC3 cells treated alone or in combination group. Two drugs resulted in more reduction of the cell viability than single treat. Two-way *T*-test results *P* < 0.05 between the two groups. (**B**) Increased levels of cleaved Caspase-3/PARP were detected in LNCaP and PC3 cells with 24 hrs of Rad001 and ATO. What's more, higher activities of Caspase-3/7 were observed in LNCaP and PC3 cells with 24 hrs treatment of Rad001 and ATO combination (*P* < 0.05 between the two groups). (**C**) Flow cytometry analysis of apoptosis with Annexin-V and 7-AAD staining. Right (top and bottom) is Annexin-V positive cells. A significant increase of percentage of apoptotic cell was observed with Rad001 and ATO combination treatment (*P* < 0.05).

The trypan blue (TB) assay demonstrated that there was more reduction of the live cells (TB negative) in both LNCaP and PC3 cells (Figure 4A), suggesting that there was the significant decrease in cell viability with combination treatment. The apoptotic proteins were semi-quantitatively detected by western blot. When PC3 and LNCaP cells with combination treatment for 24 hrs, the cleaved form of PARP was more up-regulated (Figure 4B), compared with alone treatment. What's more, the cleaved form of Caspase-3 and caspase-3/7 activity were both induced with the combination treatment (Figure 4B), suggesting combination treatment activating the apoptotic pathway. In order to quantification percent of apoptotic cells, the flow cytometric assay was performed. The Figure 4C showed combination of ATO and Rad001 may induced significantly more percentage of both early and late apoptotic cells, compared with alone compound treatment. Taking together, the combination of ATO and Rad001 led to the synergistic cytotoxicity in PC3 and LNCaP cells via induction of apoptosis.

### Combination of ATO and Rad001 synergize to induce autophagy among prostate cancer PC3 and LNCaP cell lines

**Figure 5:**
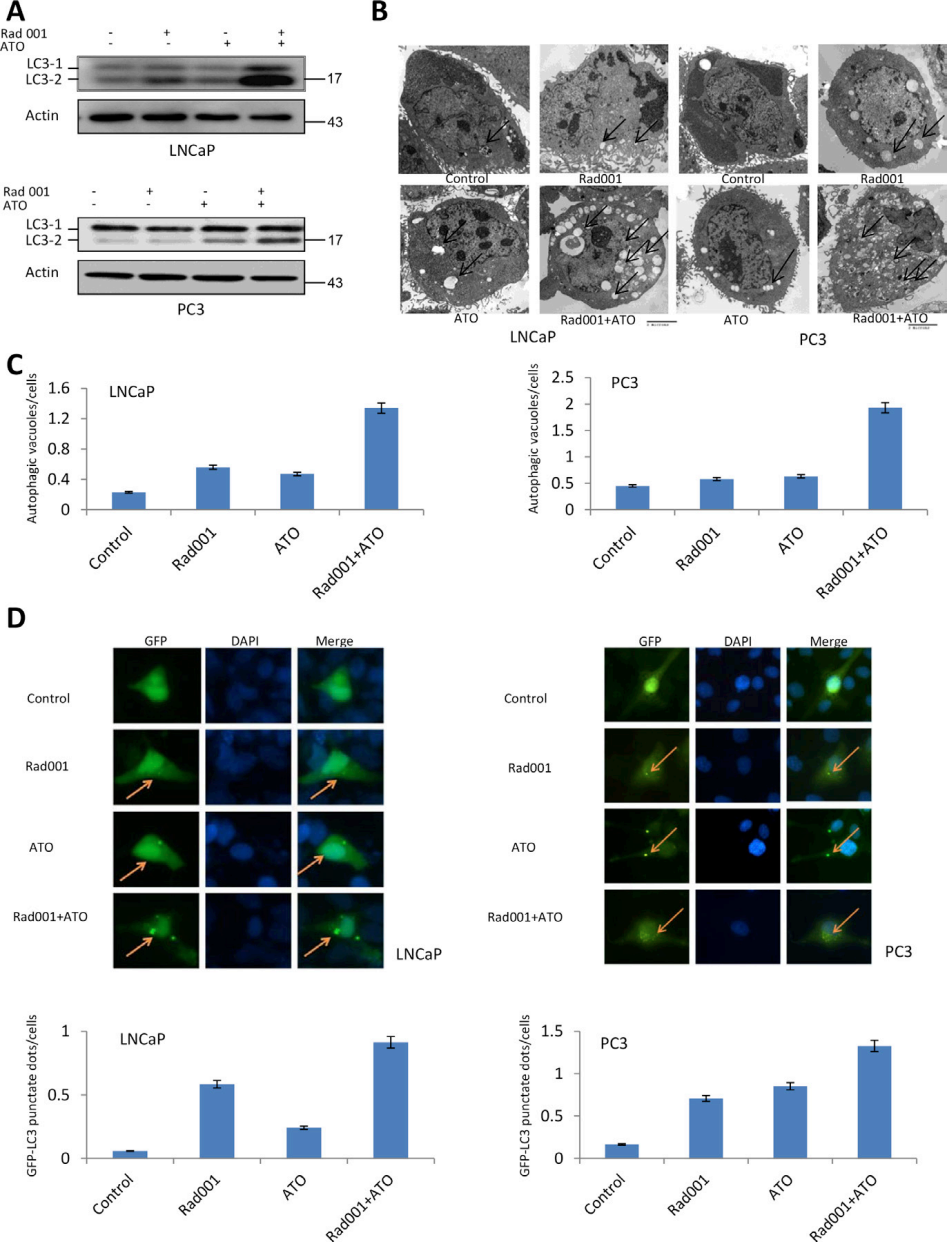
Rad001 and ATO combination synergistically induced autophagy in LNCaP and PC3 cells For LNCaP cells, they were treated DMSO (control), Rad001 (0.37 μM), ATO (11.19 μM), and their combination, for 24 hrs. For PC3 cells, they were treated DMSO (control), Rad001 (0.35 μM), ATO (12.00 μM), and their combination, for 24 hrs. (**A**) Upregulation of LC3-2 in the LNCaP and PC3 cells treated with combination of two compounds. Lysates and immunoblot was processed as in Figure 5A. (**B**) More autophagosomes (arrows) were observed under the electron microscopic images from cells with Rad001/ATO than each group alone. (**C**) The number of autophagic vacuoles/cells was quantified in cells treated with control, Rad001, ATO, and their combination. 30 cells were counted in each group. (**D**) Autophagosome analysis through GFP-LC3 expression. LNCaP and PC3 cells were transfected with GFP-LC3 plasmid, and then the cells were treatment with single compound or combination compounds. The cells were then fixed with paraformaldehyde and visualized with epifluorescence. Yellow arrows indicate the punctate pattern of GFP-LC3, representative of autophagosome, and nuclei were visualized through DAPI staining. The number of GFP-LC3 punctuate dots/cells was quantified with 50 GFP+ cells in each group.

The enhanced transformation from LC3-1 to LC3-2 is considered as the induction of autophagy. The combination treatment induced more LC3-2 conversion compared with ATO or Rad001 alone treatment (Figure 5A), suggesting the autophagy may be synergistically induced by the combination treatment. Autophagosomes is characteristic of multiple or double membrane layers structure. The Figure 5B showed the number of vacuoles cells were more obviously increased with combination of ATO and Rad001 treatment (Figure 5B), suggesting the autophagy was synergistically induced. The Figure 5C showed the quantification increase.

**Figure 6:**
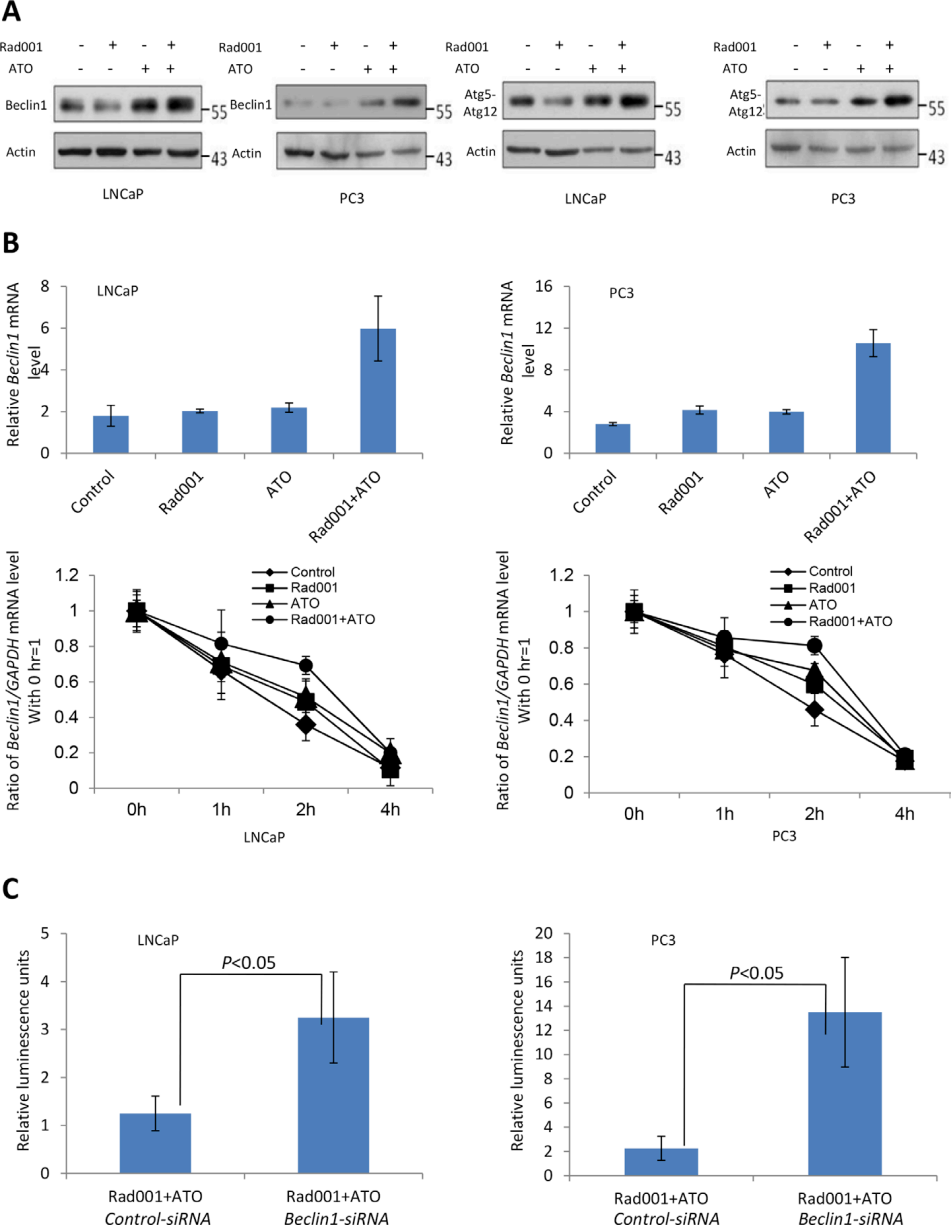
Regulation of autophagy proteins in response to combination of Rad001 and ATO For LNCaP cells, they were treated DMSO (control), Rad001 (0.37 μM), ATO (11.19 μM), and their combination, for 24 hrs. For PC3 cells, they were treated DMSO (control), Rad001 (0.35 μM), ATO (12.00 μM), and their combination, for 24 hrs. (**A**) Atg5-12 conjugates and Beclin1 were induced in response to the treatment of two compounds. (**B**) *Beclin 1* mRNA was more significantly up-regulated by the combination treatment through quantitative RT-PCR analyses in prostate cancer LNCaP and PC3 cell lines. *Beclin1* mRNA stability significantly increased in response to the combination treatment of the two compounds. RNA was extracted at the indicated time, and quantitative RT-PCR was used to measure the relative *Beclin1* mRNA level compared to that of *GAPDH*. (**C**) Beclin1 is critical for the autophagic death induced by the combination of two compounds. LNCaP and PC3 cells were transfected with control siRNA or that for *Beclin1*. 24 hrs later cells were treated with combination of Rad001 and ATO, and cell survival was gauged with ATP measurement after 24 hrs of combination treatment.

If the cells expressed the GFP-LC3, the autophagosomes are characterized by the clustered on the membrane vesicles, ring structure, etc, under the fluorescent microscope. The PC3 and LNCaP cells with combination treatment were both induced more punctuate GFP-LC3 positive vesicles (Figure 5D). The Atg5-atg12 conjugate is induced in both LNCaP and PC3 cell lines with combinatorial compounds treatment (Figure 6A). Taking together, the autophagy is synergistically induced by the combination of ATO and Rad001 treatment in prostate cancer PC3 and LNCaP cell lines, which may result in cell toxicity.

### Combination of ATO and Rad001 increased the Beclin1 expression

Beclin1 (Atg6) plays an important role in the autophagy pathway. Figure 6A showed the combination treatment induced the Beclin1 level to up-regulate. The RT-PCR analysis showed the increase of Beclin1 protein was correlated to increase of *Beclin1* mRNA level (Figure 6B). In order to probe into why the *Beclin1* mRNA increase, the half-life of *Beclin1* mRNA was examined. As shown in Figure 5B, the *Beclin1* mRNA stability was synergistically increased with the combination treatment as compared to one compound treatment, suggesting increased stability of *Beclin1* mRNA may result in the increase of *Beclin1* mRNA.

What's more, we performed small interference RNA to reduce the expression of Beclin1, and detected whether its loss may exert impacts on cell death with combination treatment. As shown in Figure 5C, the reduction of Beclin1 protein led to a significant rescue of cell death with the combinatorial compounds treatment. The percent of cell death significantly decreased with the reduction of Beclin1 protein (Figure 5C). These results suggest the increase of cell death may result from the synergistic induction of autophagy with the combination of ATO and Rad001 treatment, and Beclin1 is induced with the two compounds treatment.

### Combination of ATO and Rad001 inhibited growth of prostate cancer tumor

**Figure 7:**
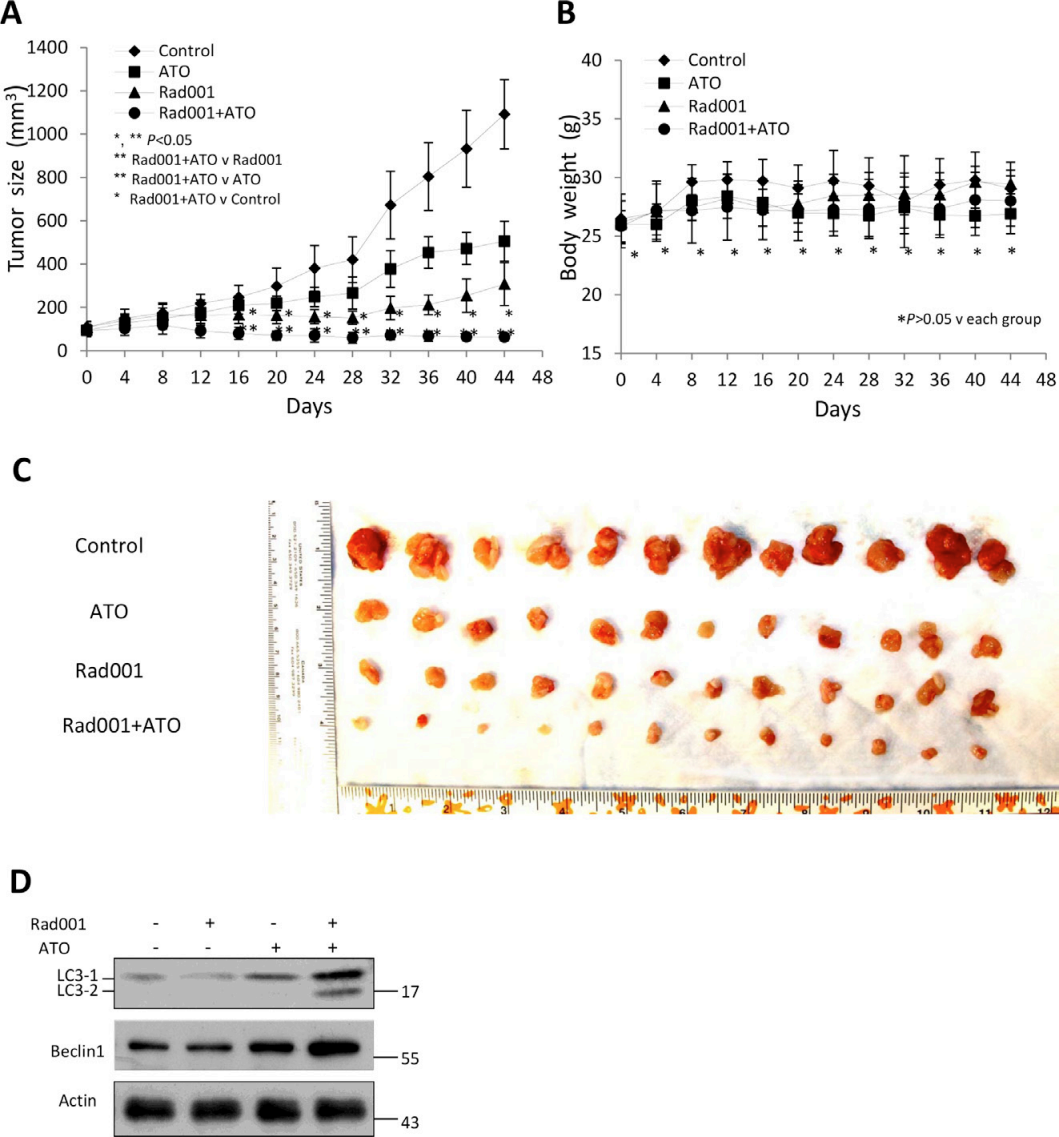
Combination of Rad001 and ATO can significantly inhibit the growth of prostate cancer LNCaP cells xenograft in a mouse model (**A**) Anti-tumor activity on LNCaP xenografts by various compounds. Mice were administered daily i.p. with Rad001 at 1 mg/kg, ATO at 2 mg/kg, and both daily (6 mice/group). The tumor sizes were measured every four days. (*P < 0.05 compared with untreated animals. **P < 0.05 compared with RAD001-treated animals or ATO-treated animals). (**B**) The mice weights were measured every four days (*P > 0.05 compared with untreated animals. **P > 0.05 compared with RAD001-treated animals or ATO-treated animals). (**C**) Tumors treated with two compounds were much smaller than single compound treatment or control. The LNCaP xenograft tumors were dissected after 44 days. (**D**) Immunoblot analysis for LC3 and Beclin1 from the lysate prepared from the xenograft.

Our studies have demonstrated that a combinatorial treatment of ATO and Rad001 can cause synergistic cell death of prostate cancer cells in the vitro. Our studies further determined if the synergism also exerts on the PCa xenograft. The ATO was administered at the dose of 2 mg/kg daily, and the Rad001 was administered at the dose of 1 mg/kg daily, which both have been commonly reported in the literature. The Figure 7A–7C demonstrated that combination of ATO and Rad001 more obviously suppressed LNCaP xenograft tumor proliferation than one compound administration (*P* < 0.05). On the other hand, body weights were not significant among each group (*P* > 0.05), suggesting a litter side effects with combination or alone treatment. Consistent with the *in vitro* studies, the molecular level of Beclin1, LC3-2 LC3-1 were all increased in the combination administration group compared with single compound group (Figure 7D).

## DISCUSSION

Endocrine therapy is one of the most common therapeutic strategy for metastatic and advanced PCa for many years. However, endocrine therapy, including the novel drugs such as enzalutamide, acetate abiraterone, etc, is palliative. A large number of patients will evolve into hormone independent or CRPC stage eventually [19, 20]. The AR amplification, mutation, splice variants, etc, are the most common mechanism of CRPC [6]. It has been reported that the AKT-mTOR pathway exerts an important role in a wide range of human cancers including PCa [21]. Xu Y et al. study showed the mTOR activity may be enhanced with androgen and autophagy also is inhibited by activation of mTOR pathway [22]. Farrow JM et al. demonstrated that PCa cells can induce autophagy to rescue the castration treatment [23]. Therefore, the autophagy may be vital of importance in the progression of PCa, and targeting its pathway may provide a novel therapeutic approach for the treatment of PCa.

Goussetis DJ et al. showed ATO can induce autophagy in the leukemia cells. Such induction of autophagy seems to require activation of the ERK pathway but AKT- mTOR pathway is unnecessary for activation of autophagy [24]. Chiu HW et al. reported the ATO induced apoptotic and autophagic cell death in human glioma cells [12]. However its therapeutic index is severely weakened by its significant clinical side effects. Our studies showed that it may synergize with Rad001, which may provides the novel therapeutic strategy. This synergistic therapeutic approach may not only reduce the side effects but increase the therapeutic effects. It also has been reported that the combination of irradiation and ATO enhances the cytotoxicity in the fibrosarcoma cancer cells via induction of both apoptosis and autophagy [11]. However, it needs a high concentration of the drug, which can cause significant clinical side effects, severely limiting the therapeutic effect of the ATO. Levine B et al. has showed that the autophagy may be induced with combination of ATO and Rad001 treatment [16]. Therefore, we detected if the combination of ATO and Rad001 synergistically induced the PCa cells death via activating the autophagy pathway.

Our studies demonstrated combination of Rad001and ATO induced PCa cells death through synergistically activation both autophagy and apoptosis. Apoptosis (programmed cell death) is considered as to be an important approach of tumor suppression. The autophagy get rid of aged or damaged materials to maintain cellular homeostasis. The stress such as chemotherapeutic compounds, nutrient starvation, hormone treatment, etc, and pathological circumstances, for example neurodegenerative disease, infectious disease, cancer, etc, can induce the autophagy [25]. Although it has been still unclear how the autophagy connect the apoptosis to kill the cells. The apoptosis can be induced by the Atg5 protein, a cleaved form of the essential autophagy. On the other hand, the autophagy may also be regulated with the Bcl-XL and Bcl-2, antiapoptotic Bcl-2 family members. The large number of studies have showed the apoptosis and autophagy could be activated simultaneously in cancer cells [26–31]. It has been reported that combination of the GX15-070 and histone deacetylase inhibitor induced the cell death through activation of both apoptosis and autophagy [32]. Although the induction of autophagy by Rad001 is not potent [33], we have demonstrated ATO can synergize with Rad001 to induce autophagic and apoptotic cell death in two prostate cancer cell lines. The mechanism of the induction of autophagy by combination of ATO and Rad001 is the result of increased stability of *Beclin1* mRNA. The combinational treatment strategy may obviously inhibit the tumorigenesis in prostate cancer LNCaP and PC3 cells, and the PC3 cells may represent the most aggressive form of late stage PCa and form of the CRPC [18]. Our studies may provide a novel therapeutic strategy for recurrence, advanced PCa, CRPC, which failed the both traditional (e.g., LHRH agonist, AR blocker) and latest (e.g., acetate abirateron, enzalutamide) forms of hormonal therapy.

In summary, we have identified that combination of two compounds induces the synergistic cell death in prostate cancer, thus potentially providing a novel therapeutic possibility for advanced and recurrence prostate cancer.

## MATERIALS AND METHODS

### Materials

LNCaP and PC3 cell lines were obtained from the American Type Culture Collection (ATCC); FBS, RPMI medium 1640, Dulbecco's modified Eagle's medium (DMEM), sodium pyruvate, L-glutamine, Penicillin, and Streptomycin were obtained from Hyclone; Rad001 and ATO were from Sigma; CellTiter assay was from Promega; Caspase-3/7 Assay Kit was from Anaspec; Annexin V apoptosis detection Kit was from eBioscience; Dharmafect transfection reagent was from Thermo Scientific Life Science; Lipofectamine 2000 transfection reagent was from Invitrogen; *Beclin1*-siRNA (5′-rGrGrArArUrGrGrArArUrGrArrGrArUrUrArA-3′, 5′-rArGrCr ArGrCrArUrUrArArUrCrUrCrArUrU-3′) and *control*-siRNA (5′-rGrArArArArArCrUrCrArUrArUrArArArUr Cr-3′, 5′-rGrUrGrGrGrGrCrGrA rUrUrUrArUrArUrGrA-3′) were from IDT; RT-PCR primers for *Beclin1*, 5′-GGCC AATAAGATGGGTCTGA-3′ and 5′-CTGCACACAG TCCAGGAAAG-3′; for *GAPDH* 5′-CATGGGTGTGA ACCATGAGA-3′ and 5′-CAGTGATGGCATGGACT GTG-3′, were from Valuegene. Rabbit anti-LC-3 polyclonal antibody was from GenScript; Rabbit anti-Beclin1, Rabbit anti-cleaved Caspase-3 and Rabbit anti-PARP1 polyclonal antibodies were from Cell Signaling; Rabbit anti-Atg5-Atg12 monoclonal antibody was from Epitomics; Mouse anti-β-Actin antibody was from Sigma; Matrigel was from BD Biosciences.

### Cell culture

The LNCaP cells were maintained in RPMI 1640 supplemented with 10% fetal bovine serum (FBS) and Penicillin-Streptomycin, and the PC3 cells were maintained in the Dulbecco's modified Eagle's medium (DMEM) containing 10% fetal bovine serum (FBS), Penicillin-Streptomycin, L-glutamine, Na^+^pyruvate. Cells were grown at 37°C with 5% CO_2_ humidity.

### Bright filed images of cells

Prostate cancer LNCaP and PC3 cells were seeded in 10 cm dishes and allowed to attach overnight. The LNCaP cells were treated with DMSO (control), Rad001 (0.37 μM), ATO (11.19 μM), or a combination of Rad001 (0.37 μM) and ATO (11.19 μM), respectively. PC3 cells were treated with DMSO (control), Rad001 (0.35 μM), ATO (12.00 μM), or a combination of Rad001 (0.35 μM) and ATO (12.00 μM), respectively. After 24 hrs treatment, the bright field images of cells were observed under the microscope (Eclipse 90i slide scope) with ×10 lens.

### Matrigel colony formation assay

Equal numbers of cells (12 × 10^3^) (50% Matrigel/Medium) were seeded into the 24-well plate. After 24 hrs the cells were treated with DMSO, Rad001, ATO, or a combination of Rad001 and ATO, respectively. The images were captured at 10× magnification with microscope (Eclipse 90i slide scope). The colonies in 6 fields at 10× magnifications were counted and reported as the average number of colonizes at the 14^th^ day.

### Relative cell luminescence units, cell viability and combination index

Equal numbers of cells (3 × 10^3^) were seeded into 96-well plate and maintained with the normal medium. After 48 hrs, LNCaP cells were treated with DMSO (control), Rad001 (0.37 μM), ATO (11.19 μM), or a combination of Rad001 (0.37 μM) and ATO (11.19 μM), respectively. PC3 cells were treated with DMSO (control), Rad001 (0.35 μM), ATO (12.00 μM), or a combination of Rad001 (0.35 μM) and ATO (12.00 μM), respectively. Three replicates were plated for each treatment group. The relative cell luminescence units were measured on after 24 hrs compound treatment. The LNCaP and PC3 cells were also treated with compounds for 24 hrs and analyzed with Trypan Blue (TB) staining. The cells were seeded into the 96-well plate at 3 × 10^3^ cells per well. After 48 hrs, LNCaP cells were treated with DMSO, 0.047 μM to 0.746 μM Rad001 (*X*-axis) for 24 hrs, 1.399 μM to 22.380 μM ATO (*Y*-axis) for 24 hrs or a combination of the two for 24 hrs. The PC3 cells were treated with DMSO, 0.180 μM to 2.80 μM Rad001 (*X*-axis) for 24 hrs, 6.00 μM to 98.00 μM ATO (*Y*-axis) for 24 hrs or a combination of the two for 24 hrs. Three replicates were plated for each treatment group. The relative cell luminescence unit was measured with the Promega CellTiter Glo (Madison, USA) assay. The compound-interactions were analyzed with *Calcusyn* software (version2.1, Biosoft) to determine the combination index (CI) for the combinatorial treatment of Rad001 and ATO.

### GFP-LC3 analysis

The cells were transfected with GFP-LC3 plasmid using Lipofectamine^TM^ 2000 transfection reagent. After 24 hrs, the medium was changed, and the LNCaP cells were treated with DMSO (control), Rad001 (0.37 μM), ATO (11.19 μM), or a combination of Rad001 (0.37 μM) and ATO (11.19 μM), respectively, for 24 hrs. The PC3 cell lines were treated with DMSO (control), Rad001 (0.35 μM), ATO (12.00 μM), or a combination of Rad001 (0.35 μM) and ATO (12.00 μM), respectively, for 24 hrs. The cells were then fixed in 4% paraformaldehyde for 30 min. The cells were washed twice with PBS and stained with DAPI, and observed under a fluorescence microscope (Eclipse 90i slide scope) with ×40 lens.

### Transmission electron microscopy

Prostate cancer LNCaP and PC3 cells were seeded in 10 cm dishes and allowed to attach overnight. The cells were treated with DMSO, Rad001, ATO and combination of Rad001 and ATO for 24 hrs. Cells were fixed in ice-cold 2.5% electron microscopy grade glutaraldehyde (in 0.1 mol/l PBS (pH 7.3)), rinsed with PBS, postfixed in 1% osmium tetroxide with 0.1% potassium ferricyanide, dehydrated through a graded series of ethanol (30–90%), and embedded in Epon. Semithin sections (300 nm) were cut using a Reichart Ultracut (Leica Microsystems Inc., Chicago, USA), stained with 0.5% toluidine blue, and examined under a light microscope. Ultrathin sections (65 nm) were stained with 2% uranyl acetate and Reynold's lead citrate and examined using JEOL 1210 transmission electron microscope.

### Protein analysis

The cultured cells were washed with cold PBS and lysed with lysis buffer (20 mM KCl, 150 mM NaCl, 1% NP-40, 50 mM NaF, 50 mM TrisHCl, pH7.5, 1 mM DTT, 1 mM EGTA, 1 × Protease Inhibitor, 10% Glycerol) for 30 min on ice. The cells were centrifuged for 15min at 4°C. The protein concentration in the supernatant was determined with the Bradford assay (Bio-Rad). Equal amount of protein was loaded on 8%, 15% SDS-polyacrylamide gels and transferred to polyvinylidene fluoride (PVDF) membrane. The membrane was blocked with nonfat dry milk for 30 min, incubated with primary antibody in nonfat dry milk overnight, washed with PBS for 30 min, incubated with secondary antibody for 1 hrs, washed with PBS/0.1%Tween20 for 1 hrs and detected with enhanced chemiluminescence (Pierce).

### Caspase-3/7 activity analysis

Equal number of the LNCaP and PC3 cells was seeded into the 96 wells plates. After 24 hrs LNCaP cells were treated with DMSO (control), Rad001 (0.37 μM), ATO (11.19 μM), or a combination of Rad001 (0.37 μM) and ATO (11.19 μM), respectively, for 24 hrs. PC3 cell lines were treated with DMSO (control), Rad001 (0.35 μM), ATO (12.00 μM), or a combination of Rad001 (0.35 μM) and ATO (12.00 μM), respectively, for 24 hrs. The caspase-3/7 activity was measured by the SensoLyte Homogeneous AMC Caspase-3/7 Assay kit after reaction for 1 hrs. All the experiments were carried out in the triplicates.

### Analysis of apoptosis

After the cells were treated the DMSO, Rad001, ATO or a combination of Rad001 and ATO for 24 hrs, the LNCaP and PC3 cells were collected. The apoptosis was quantified by FACS analysis (BD FACSDiva™ Software v6) with Annexin-V/7-AAD staining following the manufacture's guidelines. The percentage of Annexin V positive cells was analyzed by FlowJo (Version 7.6.4).

### Quantitative RT-PCR

The LNCaP and PC3 were treated with compounds for 24 hrs and total RNA was purified from the cells with Fermentas Gene RNA purification Kit. Equal amounts of RNA were reverse transcribed by reverse transcriptase (Fermentas) according to the manufacturer's instruction. Quantitative real-time PCR was performed using SA Biosciences RT^2^ Real-time^TM^ SYBR Kit with the following parameters: 15 μl, 95°C for 8min for one cycle followed by 43 cycles of 95°C for 15”/60°C for 60”.

### Small interfering RNA transfection

3 × 10^3^ of cells were seeded into the 96 wells plates and transfected with siRNA (100 nM) by the Dharmafect general transfection reagent. The combination of Rad001 and ATO was added to the cells for 24 hrs after transfection followed by cell viability measurement. To determine the knockdown efficiency of the siRNA, LNCaP and PC3 cells were seeded into the 12-wells plate followed by transfection with *Beclin1*-siRNA and *Control*-siRNA, respectively, and collected after another 24 hrs.

### *Beclin1* mRNA half-life analysis

To determine the metabolic stability of *Beclin1* mRNA, the LNCaP and PC3 cells were treated with DMSO, Rad001, ATO and its combination for 24 hrs. Actinomycin D was added at a final concentration of 10 μg ⁄mL to inhibit transcription. After 0, 1 h, 2 h and 4 h, cells were collected and washed with PBS, and total RNA was extracted. Relative levels of mRNAs were determined by real-time PCR and normalized to mRNA of *GAPDH*. All the experiments were performed in triplicates.

### Prostate cancer xenograft

Six-week-old SCID (severe combined immuno deficiency) mice were obtained from the Anhui Medical University Division of Laboratory Animal Medicine. All the mice were inoculated with 100 μl (50% Matrigel/PBS) LNCaP cells suspension (6 × 10^6^) to either dorsal flank with 25-gauge syringe. When the tumor size reached between 50 and 100 mm^3^, the mice were randomly divided into control, Rad001, ATO and combination group, with 6 mice per group. The Rad001 and ATO was delivered intraperitonealy 1 mg/kg/day and 2 mg/kg/day, respectively. The tumor sizes were measured every 4 day with a caliper. These tumor measurements were converted to tumor volume using the formula (V = 0.52 × L × W^2^), where W and L are the smaller and larger diameters. Animal body weight was also weighed the same day when the tumors were measured. At the 44th day, mice from four groups were all sacrificed; tumors were dissected and collected. All the animal experiments were conducted according to the protocol approved by the Anhui Medical University Animal Research Committee (ARC).

### Statistical analysis

The normalized isobologram analysis and bars were performed with *Calcusyn* software (version 2.1, Biosoft) and Microsoft Excel 2007, respectively. With median effects model, described by Chou [34] et.al, the multiple compound dose-effect calculations were performed. Combination index (CI) values of < 0.9, > 0.9 and < 1.2, > 1.2 was considered as synergistic, additive, antagonistic effect, respectively. Statistical analysis was performed by two-sided *t* test. *P* value less than 0.05 was considered as the statistically significant. The statistical significance of the tumor volumes of differences between treatment and control groups mice were determined by One-way ANOVA followed by the Dunnett's test. Statistical analyses on body weight were performed by One-way ANOVA followed by Tukey's test. The level of significance was set at *P* < 0.05. Statistical calculations were performed with the SPSS version 13.0.

## Abbreviations

PCa: Prostate cancer
AR: Androgen receptor
PSA: Prostatic specific antigen
ATO: Arsenic trioxide
CRPC: Castration resistant prostate cancer
APL: Acute promyelocytic leukemia
Trypan blue: TB
SCID: Severe combined immunodeficiency

## References

[1] Wang W, Yang M, Kenfield SA, Hu FB, Stampfer MJ, Willett WC, Fuchs CS, Giovannucci EL, Bao Y (2016). Nut consumption and prostate cancer risk and mortality. Br J Cancer.

[2] Siegel RL, Miller KD, Jemal A (2016). Cancer statistics, 2016. CA Cancer J Clin.

[3] Miller KD, Siegel RL, Lin CC, Mariotto AB, Kramer JL, Rowland JH, Stein KD, Alteri R, Jemal A (2016). Cancer treatment and survivorship statistics, 2016. CA Cancer J Clin.

[4] Shahabi A, Lewinger JP, Ren J, April C, Sherrod AE, Hacia JG, Daneshmand S, Gill I, Pinski JK, Fan JB, Stern MC (2016). Novel gene expression signature predictive of clinical recurrence after radical prostatectomy in early stage prostate cancer patients. Prostate.

[5] Hansen AF, Sandsmark E, Rye MB, Wright AJ, Bertilsson H, Richardsen E, Viset T, Bofin AM, Angelsen A, Selnæs KM, Bathen TF, Tessem MB (2016). Presence of TMPRSS2-ERG is associated with alterations of the metabolic profile in human prostate cancer. Oncotarget.

[6] Wadosky KM, Koochekpour S (2016). Molecular mechanisms underlying resistance to androgen deprivation therapy in prostate cancer. Oncotarget.

[7] Knudsen KE, Scher HI (2009). Starving the addiction: new opportunities for durable suppression of AR signaling in prostate cancer. Clin Cancer Res.

[8] Long Z, Chen B, Liu Q, Zhao J, Yang Z, Dong X, Xia L, Huang S, Hu X, Song B, Li L (2016). The reverse-mode NCX1 activity inhibitor KB-R7943 promotes prostate cancer cell death by activating the JNK pathway and blocking autophagic flux. Oncotarget.

[9] Zhu W, Hu X, Xu J, Cheng Y, Shao Y, Peng Y (2015). Effect of PI3K/Akt Signaling Pathway on the Process of Prostate Cancer Metastasis to Bone. Cell Biochem Biophys.

[10] Carracedo A, Baselga J, Pandolfi PP (2008). Deconstructing feedback-signaling networks to improve anticancer therapy with mTORC1 inhibitors. Cell Cycle.

[11] Chiu HW, Lin JH, Chen YA, Ho SY, Wang YJ (2010). Combination treatment with arsenic trioxide and irradiation enhances cell-killing effects in human fibrosarcoma cells in vitro and in vivo through induction of both autophagy and apoptosis. Autophagy.

[12] Chiu HW, Ho YS, Wang YJ (2011). Arsenic trioxide induces autophagy and apoptosis in human glioma cells in vitro and in vivo through downregulation of survivin. J Mol Med (Berl).

[13] Zhang S, Ma C, Pang H, Zeng F, Cheng L, Fang B, Ma J, Shi Y, Hong H, Chen J, Wang Z, Xia J (2016). Arsenic trioxide suppresses cell growth and migration via inhibition of miR-27a in breast cancer cells. Biochem Biophys Res Commun.

[14] Wang X, Li D, Ghali L, Xia R, Munoz LP, Garelick H, Bell C, Wen X (2016). Therapeutic Potential of Delivering Arsenic Trioxide into HPV-Infected. Nanoscale Res Lett.

[15] Kuma A, Hatano M, Matsui M, Yamamoto A, Nakaya H, Yoshimori T, Ohsumi Y, Tokuhisa T, Mizushima N (2004). The role of autophagy during the early neonatal starvation period. Nature.

[16] Levine B (2007). Cell biology: autophagy and cancer. Nature.

[17] Janssen T, Kiss R, Dedecker R, Petein M, Pasteels JL, Schulman C (1995). Influence of dihydrotestosterone, epidermal growth factor, and basic fibroblast growth factor on the cell kinetics of the PC3, DU145, and LNCaP prostatic cancer cell lines: relationship with DNA ploidy level. Prostate.

[18] Tai S, Sun Y, Squires JM, Zhang H, Oh WK, Liang CZ, Huang J (2011). PC3 is a cell line characteristic of prostatic small cell carcinoma. Prostate.

[19] Zhang W, Wu TY, Chen Q, Shi XL, Xiao GA, Zhao L, Xu CL, Zhou T, Sun YH (2016). Indirect comparison between abiraterone acetate and enzalutamide for the treatment of metastatic castration-resistant prostate cancer: a systematic review. Asian J Androl.

[20] Seisen T, Roupret M, Gomez F, Malouf GG, Shariat SF, Peyronnet B, Spano JP, Cancel-Tassin G, Cussenot O (2016). A comprehensive review of genomic landscape, biomarkers and treatment sequencing in castration-resistant prostate cancer. Cancer Treat Rev.

[21] Chen H, Zhou L, Wu X, Li R, Wen J, Sha J, Wen X (2016). The PI3K/AKT pathway in the pathogenesis of prostate cancer. Front Biosci (Landmark Ed).

[22] Xu Y, Chen SY, Ross KN, Balk SP (2006). Androgens induce prostate cancer cell proliferation through mammalian target of rapamycin activation and post-transcriptional increases in cyclin D proteins. Cancer Res.

[23] Farrow JM, Yang JC, Evans CP (2014). Autophagy as a modulator and target in prostate cancer. Nat Rev Urol.

[24] Goussetis DJ, Altman JK, Glaser H, McNeer JL, Tallman MS, Platanias LC (2010). Autophagy is a critical mechanism for the induction of the antileukemic effects of arsenic trioxide. J Biol Chem.

[25] Mizushima N, Levine B, Cuervo AM, Klionsky DJ (2008). Autophagy fights disease through cellular self-digestion. Nature.

[26] Lv L, Li D, Zhao D, Lin R, Chu Y, Zhang H, Zha Z, Liu Y, Li Z, Xu Y, Wang G, Huang Y, Xiong Y (2011). Acetylation targets the M2 isoform of pyruvate kinase for degradation through chaperone-mediated autophagy and promotes tumor growth. Mol Cell.

[27] Rubinstein AD, Eisenstein M, Ber Y, Bialik S, Kimchi A (2011). The autophagy protein Atg12 associates with antiapoptotic Bcl-2 family members to promote mitochondrial apoptosis. Mol Cell.

[28] Zhou F, Yang Y, Xing D (2011). Bcl-2 and Bcl-xL play important roles in the crosstalk between autophagy and apoptosis. FEBS J.

[29] Gao P, Bauvy C, Souquere S, Tonelli G, Liu L, Zhu Y, Qiao Z, Bakula D, Proikas-Cezanne T, Pierron G, Codogno P, Chen Q, Mehrpour M (2010). The Bcl-2 homology domain 3 mimetic gossypol induces both Beclin 1-dependent and Beclin 1-independent cytoprotective autophagy in cancer cells. J Biol Chem.

[30] Lian J, Karnak D, Xu L (2010). The Bcl-2-Beclin 1 interaction in (−)-gossypol-induced autophagy versus apoptosis in prostate cancer cells. Autophagy.

[31] Liang C (2010). Negative regulation of autophagy. Cell Death Differ.

[32] Wei Y, Kadia T, Tong W, Zhang M, Jia Y, Yang H, Hu Y, Tambaro FP, Viallet J, O'Brien S, Garcia-Manero G (2010). The combination of a histone deacetylase inhibitor with the Bcl-2 homology domain-3 mimetic GX15-070 has synergistic antileukemia activity by activating both apoptosis and autophagy. Clin Cancer Res.

[33] Crazzolara R, Bradstock KF, Bendall LJ (2009). RAD001 (Everolimus) induces autophagy in acute lymphoblastic leukemia. Autophagy.

[34] Chou TC (2010). Drug combination studies and their synergy quantification using the Chou-Talalay method. Cancer Res.

